# Spatially and spectrally resolved orbital angular momentum interactions in plasmonic vortex generators

**DOI:** 10.1038/s41377-019-0136-z

**Published:** 2019-03-20

**Authors:** Jordan A. Hachtel, Sang-Yeon Cho, Roderick B. Davidson, Matthew A. Feldman, Matthew F. Chisholm, Richard F. Haglund, Juan Carlos Idrobo, Sokrates T. Pantelides, Benjamin J. Lawrie

**Affiliations:** 1 Center for Nanophase Materials Science, Oak Ridge National Laboratory, Oak Ridge, TN 37831 USA; 2 Klipsch School of Electrical and Computer Engineering, New Mexico State University, Las Cruces, NM 88003 USA; 3 Department of Physics and Astronomy, Vanderbilt University, Nashville, TN 37235 USA; 4 Quantum Information Science Group, Oak Ridge National Laboratory, Oak Ridge, TN 37831 USA; 5 Materials Science and Technology Division, Oak Ridge National Laboratory, Oak Ridge, TN 37831 USA; 6 Department of Electrical Engineering and Computer Science, Vanderbilt University Nashville, Nashville, TN 37235 USA; 7 Present Address: Chemistry Division, U.S. Naval Research Laboratory, Washington, D.C. 20375 USA

**Keywords:** Metamaterials, Nanophotonics and plasmonics, Scanning electron microscopy, Optical spectroscopy

## Abstract

Understanding the near-field electromagnetic interactions that produce optical orbital angular momentum (OAM) is crucial for integrating twisted light into nanotechnology. Here, we examine the cathodoluminescence (CL) of plasmonic vortices carrying OAM generated in spiral nanostructures. The nanospiral geometry defines a photonic local density of states that is sampled by the electron probe in a scanning transmission electron microscope (STEM), thus accessing the optical response of the plasmonic vortex with high spatial and spectral resolution. We map the full spectral dispersion of the plasmonic vortex in spiral structures designed to yield increasing topological charge. Additionally, we fabricate nested nanospirals and demonstrate that OAM from one nanospiral can be coupled to the nested nanospiral, resulting in enhanced luminescence in concentric spirals of like handedness with respect to concentric spirals of opposite handedness. The results illustrate the potential for generating and coupling plasmonic vortices in chiral nanostructures for sensitive detection and manipulation of optical OAM.

## Tiny spirals make twisted light

Nano-sized spiral structures developed by researchers in the US provide an excellent platform to generate and detect light beams carrying angular momentum. This so-called twisted light, with a helical wave shape, could be used to manipulate tiny objects in future devices such as quantum computers. Jordan Hachtel at Oak Ridge National Laboratory, Tennessee and co-workers fabricated ‘nanospirals’ on silver-coated silicon nitride membranes, and bombarded the samples with a high-energy electron beam from a scanning transmission electron microscope (STEM). The beam excites a coupled light-electron surface state called a plasmonic vortex, which emits twisted light. Crucially, the STEM technique can observe the luminescence coming from the vortex with nanoscale spatial resolution across the entire visible-light regime.

## Introduction

Light carrying orbital angular momentum (OAM) is a topic of broad current interest^1–6^. The helical wavefront of optical OAM modes can both detect and alter the angular momentum of micro- and nanoscale structures and provide novel routes to the optical manipulation of matter^7–10^. The additional degrees of freedom provided by an orthonormal OAM basis set enable large-scale multiplexing of both classical and quantum communications and provide a framework for emerging studies in quantum information science^11–16^. It has also been proposed that OAM modes could be used to interact with chiral molecules, which play a critical role in biological and chemical processes^17–21^. Many of these applications are centered around nanotechnology; thus, the integration of optical OAM into nanoscale devices is now a critical research thrust^22–24^.

Surface plasmons are routinely used to manipulate light at the nanoscale. Research in this area has driven the development of metasurfaces and asymmetric plasmonic nanostructures that enable on-chip generation and control of OAM^25–31^. Nanoscale OAM has been characterized in plasmonic nanostructures by scanning near-field techniques^32–35^ and time-resolved photoemission electron microscopy (TR-PEEM)^36^, highlighting the broad potential for near-field applications of OAM modes in nanotechnology. However, previous explorations of OAM in plasmonics have all employed monochromatic, resonant optical excitation determined by the geometry of the plasmonic structures. Thus, a nanoscale description of the dispersion in plasmonic vortices with high spectral and spatial resolution is a critical step in developing proposed advanced applications for nanophotonic OAM states with no preconceptions about the spatial and spectral properties of the plasmonic vortex^37^.

In this paper, we investigate the cathodoluminescence (CL) response of plasmonic vortices generated in spiral nanostructures by the fast electron beam of a scanning transmission electron microscope (STEM). Archimedean spiral channels present an ideal testbed for the near-field study of OAM because they can be designed to produce plasmonic vortices with geometry-dependent OAM in structures of submicron dimensions. The relativistic electrons in the STEM initiate an impulsive excitation within an ~1 fs period localized to a spot size of ~1 nm diameter, allowing for the analysis of complex optical phenomena in plasmonic materials with high spatial and spectral resolution^38–44^. The CL generated by this electron beam excitation permits a simultaneous examination of the near-field plasmonic phase and amplitude across a broad spectral range in the nanoscale vortex generator. As a result, coupling of the plasmonic vortices to other chiral nanostructures can be observed in the enhanced CL of coupled plasmonic structures, where both the vortex generator and the secondary nanostructure have the same handedness, enabling the direct detection of spatially and spectrally resolved OAM interactions.

## Results

An Archimedean spiral is described by the polar equation, $r\left( \theta \right) = r_0 + d \cdot \frac{\theta }{{2\pi }}$, where *r*_*0*_ is the initial radius, *θ* is an azimuthal coordinate in radians, and *d* is the interarm distance. Simulations have shown that plasmonic vortices with optical OAM occur in Archimedean nanospiral grooves via coherent surface plasmon polariton (SPP) interactions in the system^25^. The measured OAM of the vortex arises from the coupling the topological charge produced by the vortex-generator structure with the angular momentum of the exciting source. In the present experiment, the OAM of the plasmonic vortex is explored directly by studying the spiral with an exciting electron beam carrying zero angular momentum.

If the dimensions of the spiral are chosen such that the arm spacing, *d*, is an integer multiple, *m*, of the SPP wavelength, *λ*_SPP_, there is a 2 mπ phase offset between the beginning and end of the channel, and the composite plasmonic response of the system has the form of a Bessel function. The order, or topological charge, of the plasmonic vortex in the spiral channel is determined by the vector sum of the topological charge produced by the nanostructure geometry and the angular momentum of the exciting source. The topological charge of the spiral is defined by the ratio of interarm separation to SPP wavelength: *ℓ* = *d*/*λ*_SPP_^25^. The electromagnetic field $U_\ell (r,\theta ,z)$ of such a mode in cylindrical coordinates is therefore

1 $$U_\ell (r,\theta ,z) = e^{i\ell \theta }J_\ell (k_rr)e^{ik_zz}$$

where *J*_*ℓ*_ is an *ℓ*^th^-order Bessel function of the first kind; *k*_z_ and *k*_*r*_ are the perpendicular and radial wavenumbers, respectively; and *k*_z_^2^ + *k*_r_^2^ = *ϵk*_0_^2^, where *k*_*0*_ is the wavenumber of light in free space, and *ϵ* is the dielectric constant of the material.

**Fig. 1:**
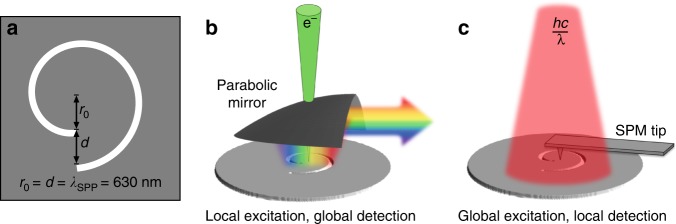
STEM-CL analysis of plasmonic vortices. **a** Schematic of the spiral channel plasmonic vortex generator, with initial radius, *r*_0_, equal to the arm separation, *d*, equal to the surface plasmon polariton (SPP) wavelength (630 nm). **b**, **c** Schematics illustrating the distinction between CL **b**, which is acquired with local excitation and global detection, and other methods **c**, which are acquired via global excitations and local detection

The advantage of STEM-CL for analyzing plasmonic vortex generators arises from the fact that it inherently involves local excitation and global detection. This contrasts with other techniques for spatially resolved analysis, such as scanning near-field optical microscopy (SNOM) or PEEM, in which global excitation is followed by local detection. The distinction between the two approaches is illustrated in Fig. 1b, c. In STEM-CL (Fig. 1b), a convergent probe creates a strongly localized excitation and the far-field, broadband CL is collected by the parabolic mirror. In contrast, techniques such as SNOM or PEEM (Fig. 1c) rely on a monochromatic beam that constantly pumps the plasmonic vortex across the whole structure, while localized changes to the electric field are detected by the scanning probe tip or electron optics. As a result, while optically driven techniques simultaneously excite many plasmons, vortex excitation and detection in STEM-CL allows for broadband exploration of plasmon interactions at the single-to-few plasmon scale, providing new insights into the dynamics of plasmonic vortices.

To access the broadband optical response of the spiral structure with nanometer spatial resolution, a hyperspectral map, or spectrum image (SI), of the spiral channel is obtained using STEM-CL. The SI is a three-dimensional data set that describes the full visible to near-infrared spectrum of the CL at each pixel sampled by the rastered electron beam. From the SI, slices of the hyperspectral response are generated in which the integrated intensity over a specific spectral range is plotted at each pixel to generate 2D maps of specific resonant responses.

**Fig. 2:**
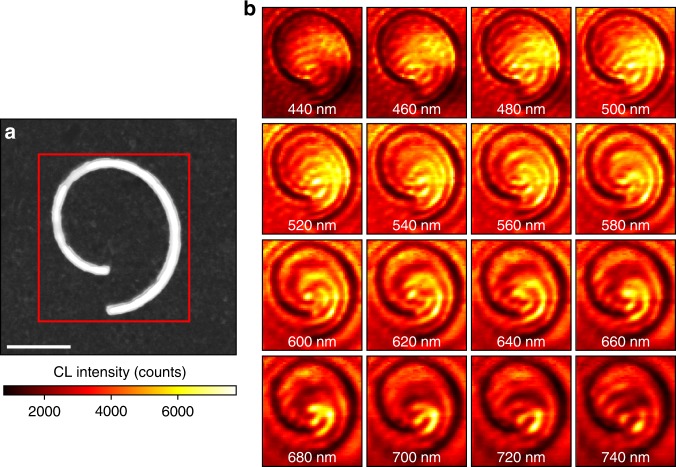
Spatially and spectrally resolved plasmonic vortices. **a** Bright-field (BF) scanning transmission electron microscopy (STEM) image of a spiral channel in a silver film. The red box marks the region for a spectrum image (SI). Scale bar = 1 μm. **b** Cathodoluminescence (CL) SI of the spiral channel. Each frame shows a 10 nm wavelength slice of the spectrum image centered at the value on the image, showing the hyperspectral response of the vortex from 440 nm to 740 nm. Scale bar = 1 μm

Figure 2a shows a bright-field (BF) STEM image of Archimedean spiral channels, with a red rectangle defining the region of interest for the SI. Figure 2b shows a frame-by-frame picture of the SI in 10-nm wavelength bins with 20-nm intervals from 440 nm to 740 nm, illustrating the combined spatial and spectral response of the plasmonic vortex generator.

At both short- and long-wavelength extremes of the SI, the CL illustrated in Fig. 2b does not exhibit a vortex response and is dominated by linear interference fringes. However, over the middle spectral range (~540–660 nm), the plasmonic vortex, consisting of two spiral arms emerging from the origin of the Archimedean spiral, is resolved. In  supplementary information (Fig. S3), the spiral arms are shown on the CL-SI slices with spiral annotations to clearly demonstrate the resolved vortex profile. Additionally, a video showing the plasmonic response of the system over the full collected spectral range is included in supplementary information and shows the vortex dispersion with a large number of narrow wavelength bins (~4 nm per bin).

**Fig. 3:**
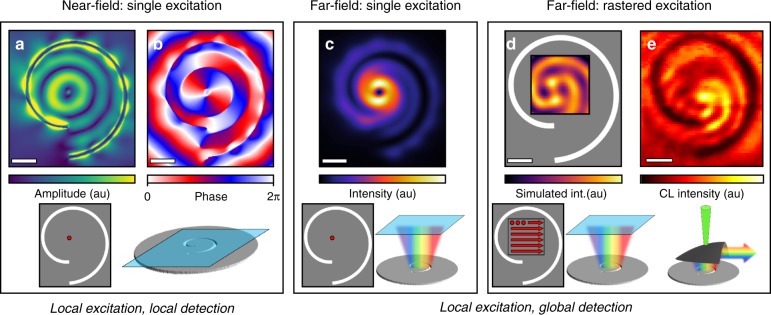
Cathodoluminescence signature of plasmonic vortex. **a**, **b** Finite-difference time-domain (FDTD) simulations of the electric-field amplitude **a** and phase **b** of a plasmonic vortex generated from a single dipole excitation at the origin of a spiral channel at a wavelength of 660 nm. **c** FDTD simulated far-field response of a plasmonic vortex transmitted through a plane 200 nm above the surface of the spiral for the same wavelength as in **a** and **b** showing the characteristic annular profile of a vortex beam. **d** The rastered and integrated far-field profile of the plasmonic vortex calculated via the far-field intensity profile from a dipole excitation is calculated for every position in a 40 × 40 array covering a 1 µm × 1 µm area around the origin of the spiral (closest direct analog to CL). **e** The CL response of the plasmonic vortex at 660 nm. In both the simulated **d** and experimental **e** rastered images, the pattern of concentric spiral arms emanating from the origin can be observed. Scale bar = 500 nm

To understand the profile of the plasmonic vortex, we carry out finite-difference time-domain (FDTD) simulations in which the fast electrons are treated as point-dipoles oriented along the beam (*z-*direction)^45^. Figure 3 shows the simulated and experimental profiles of the plasmonic vortex at a wavelength of 660 nm. Figure 3a, b shows the *z*-components of the near-field amplitude and phase, modeled, respectively, with a *z-*oriented dipole excitation at the origin of the spiral and the detector plane at the plasmonic vortex-generator surface.

The near-field amplitude simulations show the characteristic signature of an *ℓ* = 1 Bessel mode, comprising a null region at the origin with rotationally symmetric, high-intensity concentric rings filling the area between the spiral arms. The vorticity of the near-field response is contained entirely in the plasmon phase, which shows the characteristic phase singularity at the origin with a rotating phase throughout the structure.

The amplitude and phase simulations of the plasmonic vortex shown in Fig. 3a, b correspond to an experiment with local excitation and local detection, which, while providing a detailed observation of the near-field structure, do not directly correspond to the local excitation and global detection CL results shown in Fig. 2. To connect the experimental CL response to the near-field simulations, we perform local excitation, global detection simulations of the generator. Figure 3c shows the calculated far-field emission profile of the plasmonic vortex shown in Fig. 3a, b. The far-field intensity here is taken as the *z*-component of the Poynting vector at a detector plane 200 nm above the surface of the spiral. The dominant far-field feature is the well-known Laguerre-Gauss profile associated with vortex beams.

The simulations in Fig. 3c represent a local excitation, global detection experiment; however, the spatial resolution in STEM corresponds to a scalar signal correlated to a highly localized electron probe position, as opposed to a spatially resolved image collected in the far field. In fact, in STEM-CL, spatial aberrations induced by the small form-factor parabolic mirror preclude direct far-field imaging of the CL profile in this case.

To formulate a directly comparable simulation, we integrate the emitted intensity from far-field profiles such as the one shown in Fig. 3c from a series of *z-*dipole excitations across a 40 × 40 scanning grid covering a 1 μm × 1 μm area around the origin of the plasmonic vortex generator. This approach, although computationally expensive, presents a stronger physical analogy to STEM-CL, as it captures the effect of multiple independent local excitations of SPPs from a rastered, local excitation source, such as that found in the focused electron beam excitation and global signal collection characteristic of CL spectroscopy.

The dipole-array simulation of the far field intensity (simulated CL) at a wavelength of 660 nm is shown in Fig. 3d, with the experimentally measured CL-SI frame for 660 nm in Fig. 3e. In both the 40 × 40 array simulations and the CL-SI frames, concentric spiral arms of high intensity are observed spreading out from the origin of the spiral. The spiral arms indicate the presence of vorticity in the CL signal, and since the vorticity in the plasmonic vortex is entirely contained in the near-field phase, we can determine that the spatial and spectral CL signal is directly modulated by the vortex phase.

Electron excitations in plasmonic materials are often connected to the near-field amplitude of the plasmon modes, but more generally, the loss function of the electron beam traversing the sample can be directly related to the photonic local density of states (LDOS)^46,47^,

2 $${\mathrm{\Gamma }}({\boldsymbol{R}},\hbar \omega ) = \frac{{2{\mathrm{\pi e}}^2}}{{\hbar \omega }}\tilde \rho _{\hat z}({\boldsymbol{R}},q,\hbar \omega )$$

Here, ${\mathrm{\Gamma }}({\boldsymbol{R}},\hbar \omega )$ is the loss probability per unit length, at position ***R*** and energy $\hbar \omega$. The photonic density of states $\tilde \rho _{\hat z}({\boldsymbol{R}},q,\omega )$ in the $\hat z$ direction is a function of position ***R***, wavevector $q$, and excitation energy $\hbar \omega$.

Equation 2 shows how STEM-CL facilitates the analysis of complex optical structures by incorporating nanoscale optical effects and spatial variations in the strength of the measured signal. More significantly, the combination of local excitation and global detection in STEM-CL enables the collection of localized interference between different optical excitations. Here, the plasmon phase at the nanoscale is detected in the interference between the plasmon CL and isotropic transition radiation (TR).

As shown in Fig. 2, the STEM-CL response at wavelengths far from the OAM mode resembles linear interference fringes rather than a chiral plasmonic vortex. Similar interference patterns have been observed previously in the scanning electron microscope (SEM) CL from linear plasmonic gratings and attributed to the interference between SPPs and TR generated by fast electrons impinging on the metal surface, a phenomenon not included in the near-field amplitude. Kuttge et al. have modeled the interference between TR and SPPs as ref. ^48^

3 $$I_{{\rm CL}} = \mathop {\int}\nolimits_{{\!\!\!\rm mirror}}^{} {{\rm d}{\mathrm{\Omega }}|{\rm A}_{{\rm SPP}}{\rm S}\left( {\mathrm{\Omega }} \right)e^{i\phi } + f_{{\rm TR}}\left( {\mathrm{\Omega }} \right)|^2}$$

where Ω is the angle of the emission, A_SPP_ is the SPP amplitude, S(Ω) is the normalized in-plane far-field amplitude of the SPP after being scattered by a grating, *f*_TR_ is the far-field amplitude of the transition radiation, and *ϕ* is the phase difference between the SPP and TR.

While TR is not directly included in the simulations shown in Fig. 3, the interference is still observed because the radiation from the oscillating point-dipoles in each calculation of the array gives an accurate representation of TR^48^. The calculated radiated intensity is determined by the magnitude of the Poynting vector, and hence reflects the constructive and destructive interference between the dipole radiation and plasmonic CL that defines the far-field profile. The vortex is resolved in the far-field rastered simulations but not in the near-field single-excitation simulation, demonstrating that interference in the coherent sum of TR and SPP emission is a defining feature of the CL as described by Eq. 3. Hence, the vorticity of the plasmon CL directly measures the phase of a plasmonic vortex at the nanoscale, without recourse to far-field measurements of the phase of either scattered or emitted light.

**Fig. 4:**
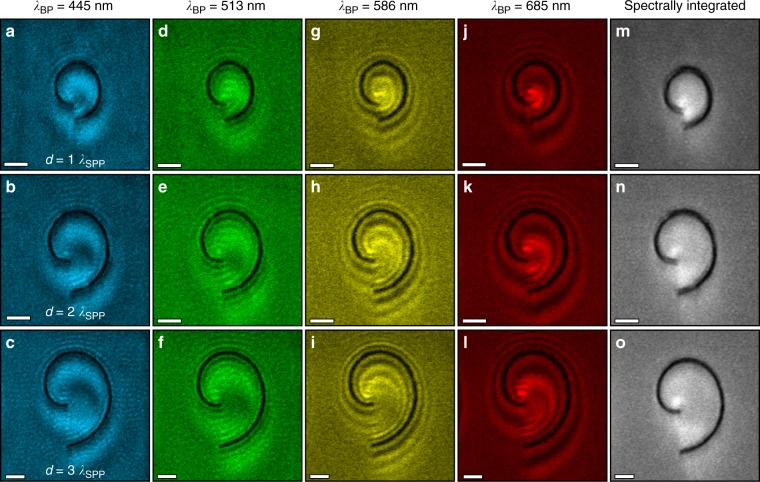
Spectrally filtered plasmon cathodoluminescence maps. **a**–**l** Band-pass filtered (BP) CL images of spiral channels with arm separations equal to *λ*_SPP_, 2 *λ*_SPP_, and 3 *λ*_SPP_. The BP filters are centered at 445 nm **a**–**c**, 513 nm **d**–**f**, 586 **g**–**i**, and 685 nm **j**–**l**, and exhibit the vortex response of the OAM mode, generated by the phase of the plasmon modes. **m**–**o** Unfiltered CL images of the same spiral channels, showing that the phase-induced effects are only detected with spectral selectivity, and cannot be seen in the spectrally integrated luminescence of the system. Scale bars = 1 μm

To illustrate the influence of plasmon phase on the detected CL signal, vortex generators with varying topological charges – that is, varying geometrical parameters – were examined using bandpass (BP) filtered CL as depicted in Fig. 4. The filter bandwidths are all 40 nm with center wavelengths at 445 nm (a–c), 513 nm (d–f), 586 nm (g–i), and 685 nm (j–l), respectively. The three structures are spiral channels with *r*_0_ *=* *λ*_SPP_, and *d* *=* *λ*_SPP_ (a, d, g, j), 2*λ*_SPP_ (b, e, h, k), and 3*λ*_SPP_ (c, f, i, l), resulting in topological charges of *ℓ* = 1, 2, and 3, respectively. The effect of the vortex phase on the CL signal is evident for all three structures in both the 586-nm and 685-nm BP-filtered CL images. As shown in Fig. 3, only the phase of the vortex shows an angular variation, while its amplitude is rotationally symmetric. Thus, the vortex response in the CL implies that the plasmon phase has a strong influence on the total detected signal.

The photomultiplier tube (PMT) detector used in the BP-filtered CL has a higher collection efficiency than the spectrometer, allowing for faster data acquisition and larger fields-of-view and enabling analysis of the plasmonic dispersion outside the spiral channel. Here, fringe patterns resulting from the interference between TR and SPP emission are wavelength- but not structure-dependent and are observed for all three structures. The fringes do not depend on the interarm spacing of the spiral because the electron beam cannot excite a plasmonic vortex outside the channel. However, the fringes are dependent on the wavelength, because different SPP wavelengths accumulate different total phase changes with respect to the TR – determined from the difference between the electron probe position and the channel edge from which SPPs radiate – and the SPP dispersion relation. The interference pattern outside the spiral further demonstrates that the plasmon phase modulates the detected signal. The CL spatial profile within the spirals depends strongly on the channel dimensions and wavelength, indicating that both phase structure and topological charge of the plasmonic vortex influence the interference patterns in Fig. 4.

The interferogram created in the coherent interaction between the TR and SPP emission measures the phase of the plasmonic vortex relative to that of the TR. Because the TR is emitted immediately when the fast electron impinges on the metal surface, the distance between the point of excitation and the SPP decay at the channel edge imparts a well-defined phase difference between the TR and SPP emission at each probe position. However, it is important to note that the phase measured in the CL signal is the coherent sum of the TR and SPP emission, rather than the direct near-field phase signature of the plasmonic vortex. If that coherent sum were not measured, the CL profile would not be sensitive to the phase of the vortex. Figure 4m–o shows unfiltered CL images of the three spirals in Fig. 4, in which the spectral response of the plasmonic vortex is integrated to yield the total luminescent intensity of the vortex. There is little spatial variation in the unfiltered CL profiles of the three spirals and no evidence of the plasmonic vortex because of the significant plasmon dispersion across the visible spectrum.

**Fig. 5:**
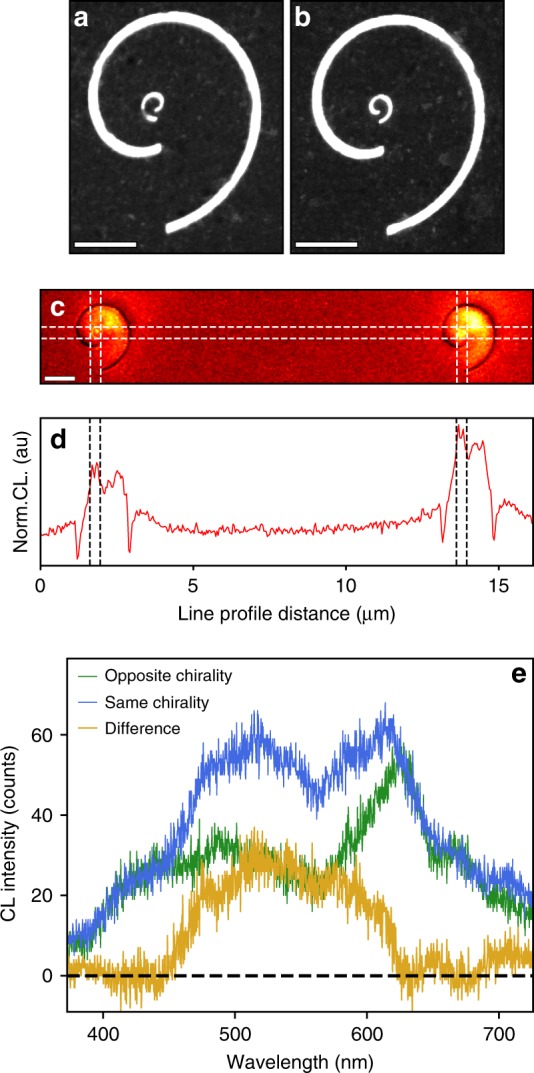
Coupling between plasmonic vortices and chiral structures. **a**, **b** BF-STEM image of two spirals (each with 2 *λ*_SPP_ arm spacing) with a secondary spiral milled at the origin of the outer spiral, one with the opposite chirality (OC) to the outer spiral **a** and the other with the same chirality (SC) as the outer spiral **b**. Scale bars = 1 μm. **c** Unfiltered CL images of the two structures (OC-left, SC-right), showing enhanced luminescent intensity from the SC structure. **d** Line profile across the inner spirals in **c**. Scale bar = 2 μm. **e** CL spectra from the center of each inner spiral, showing that the SC structure shows a broad enhancement across the visible spectrum

Finally, by experimentally characterizing the photonic LDOS, we can analyze the near-field interactions between coupled chiral nanostructures. Figure 5a, b shows BF-STEM images of two spirals, each with *d* = 2 *λ*_SPP_. The inner spiral, with a radius an order of magnitude smaller, is milled at the origin of the outer spiral. Figure 5a shows a structure in which the inner and outer spirals have opposite chiralities (OC), while on the right (Figure 5b), the two structures have the same chiralities (SC). To assess the coupling between the inner and outer spirals, the OC and SC structures are examined simultaneously with unfiltered PMT-CL imaging.

Figure 5c, d shows single unfiltered CL images (Fig. 5c) and line profiles of the CL images taken across the origin of the two inner spirals (Fig. 5d). The luminescent intensity generated in the SC structure is significantly greater than that produced by the OC structure. Since the images are spectrally integrated, the interference-driven phase effects are not observed, and the difference between the two chiral structures can therefore be directly connected to the photonic LDOS of the composite systems. Because the SPPs excited by the electron beam sample the entire geometry, excitations at every probe position within the channel experience the handedness of the outer spiral; the resulting composite response reflects that handedness and is geometrically focused at the origin of the inner spiral. The enhanced photonic LDOS integrated across the vortex-generating region indicates that the chiral plasmon outcouples more efficiently when the inner spiral has the same handedness as the outer spiral.

Furthermore, by collecting spectra from the inner spirals of the two systems, the spectral range of the luminescence enhancement can be observed. In Fig. 5e, we compare individual spectra from the inner spiral of each pair of spiral strictures. The enhanced CL intensity in the SC spirals spans a broad bandwidth in the visible range from 450 nm to 620 nm. The enhanced luminescence across this spectral region signals that the photonic LDOS for a broad bandwidth of plasmon modes has increased. We recall from Fig. 1 that the plasmonic vortex in an isolated spiral channel was also observed across a similar bandwidth, thus reinforcing the observation of enhanced coupling between plasmonic vortices and nanoscale chiral structures.

## Discussion

The STEM-CL analysis of a complex plasmonic system, such as a vortex generator, has several important implications beyond the high spatial and spectral resolution, specifically the detection of the phase structure in time-averaged, spatially resolved techniques. In photonics, the phase of optical phenomena is most often studied by overlapping the mode of interest with a separate coherent source to produce interferograms^1–3^. However, for probe techniques with nanoscale spatial resolution, such as STEM, SNOM, or PEEM, multiplexing is not so straightforward, and the capacity to directly resolve the phase is an outstanding problem. The phase can also be accessed through time-resolved measurements, and many researchers have used time-resolved SNOM and PEEM to access plasmonic phases^36,49,50^. Alternatively, in transmission electron microscopy in particular, researchers have worked to modify the phase of the incoming electron beam to access phase effects in their samples^51–53^. Such techniques are powerful, but require significant investments and modifications to standard instrumentation to achieve, while in CL the phase modulation is built-in, due to the capacity to excite multiple competing optical signals coherently with a highly localized excitation.

Furthermore, the capacity to examine chiral nanostructures, such as the Archimedean spiral, enable the direct examination of near-field OAM by producing optical vortex beams from features with nanoscale dimensions. By exciting the vortex locally, and detecting the response globally, STEM-CL characterizes the phase and amplitude of the plasmon response directly with an optimal combination of spatial and spectral resolution. The difference between electron-driven and light-driven excitations of chiral plasmon modes presents a fascinating complement to the bulk of the current literature, with recent electron microscopy results exploring spatially resolved plasmonic dichroism^54^ and yielding new insights into topics, such as holographic light generation^55^. Here, we have provided the first spatially and spectrally resolved description of the plasmonic vortex amplitude and phase by exploring the interference between SPPs and TR. Additionally, we have demonstrated enhanced plasmonic coupling between nanostructures of like chirality. The ability to experimentally examine near-field interactions of complex composite plasmonic effects, such as plasmonic vortices, opens the door to potential applications in molecular chirality detection and manipulation of twisted light in nanoscale structures.

## Materials and methods

### Sample preparation

The Archimedean spiral channels, shown schematically in Fig. 1a, are fabricated by evaporating 100 nm of Ag onto an electron-transparent SiN membrane (~50 nm thick) at a rate of 0.5 Å/s, followed by focused ion beam (FIB) milling of the spiral channel in Ag in an FEI (now Thermo Fisher - Waltham, MA, USA) Novalab 600 Dual-Beam system. The channel width is chosen to be 100 nm to avoid the redeposition of Ag during FIB milling; more details are included in supplementary information. In the spiral channel, *r*_0_ and *d* are both chosen equal to *λ*_SPP_, which is 630 nm for the vacuum/Ag/SiN multilayer structure. The nominal dimensions of the spiral channel yield a free-space wavelength of 660 nm for the OAM mode; with the interarm spacing equal to the SPP wavelength, the topological charge for the plasmonic vortex is equal to 1.

### Finite-difference time-domain simulations

To numerically compute the plasmonic response of the Archimedean spirals, we use a commercial FDTD solver, Lumerical Solutions^TM^ (Vancouver, BC, Canada). In the simulation, the computation window is enclosed by a perfectly matched layer (PML) to avoid any artificial reflection from the boundaries. The plasmonic responses of the spirals are studied under two different optical excitation types: a circularly polarized plane-wave excitation and an electric dipole (ED) excitation. For the plane-wave excitation, two linearly polarized plane-waves with a relative phase difference of *π*/2 are excited, simultaneously. To compare the measured scanning-transmission-electron-microscopy image with simulation, a broadband z-oriented ED source is used. The overall electromagnetic response of the spirals is calculated by scanning the excitation location of the ED source within a 1 µm-by-1 µm square region, centered at the origin of each spirals with a step size of 25 nm. At each excitation location, we run the simulation twice – with and without the spirals – to separate the contributions to the amplitude that are due to the dipole excitation. The time-averaged Poynting vector (*P*_*z*_) is calculated at each excitation point and plotted. The optical properties of Ag used in the calculation are obtained from Palik^56^.

### Cathodoluminescence characterization

All STEM experiments described here are performed on a VG-HB601 (company no longer in business) dedicated STEM outfitted with a 2 str solid angle parabolic mirror. The electron beam has an energy of 60 keV, resulting in an inelastic mean free path of the fast electrons in Ag of ~50 nm^57^. All acquisitions here are conducted with a probe current of 2 nA, with an average arrival time between electrons of ~100 ps. For a system with a shorter mean free path than the film thickness, usually only one or two plasmons are excited from each beam electron. Furthermore, since the plasmon lifetime is many orders of magnitude faster than the ~100 ps delay between electrons, each vortex is generated from an individual SPP excitation at the highly localized electron probe position. The SIs shown in this paper are acquired on a Princeton Instruments (Trenton, NJ, USA) Acton 2500 spectrometer equipped with a PIXIS Excellon 100BR CCD, with a dwell time of 2 s/pixel. The BP-CL images were acquired over a 256 × 256 pixel region with a Hamamatsu (Ichino-cho, Japan) R9110 Peltier cooled PMT detector with acquisition times of 160 µs per pixel.

The CL signal is free-space coupled into both the spectrometer and the PMT through a transparent window in the microscope column. For the PMT detector, a lens tube is used to connect the PMT objective to the CL port and to avoid light pollution. For the spectrometer, the entrance slit blocks external sources sufficiently to isolate the CL signal. For all microscopy, we use Gatan’s (Pleasanton, CA, USA) Digital Micrograph (DM) microscope control software package, and for all spectrometer acquisitions, we use Princeton Instruments’ WinSPEC. Band-pass filtered CL is acquired by feeding the PMT signal directly into the DM scan control, such that it can be read as a standard microscope detector signal. We use the built-in spectrum imaging tool in DM, and trigger CL acquisitions on WinSPEC from the scan control. The individual spectra acquired from WinSPEC are then transformed into a CL-SI using Python-based postprocessing.

## Supplementary information

Supplementary Materials

Supplemental Materials: Video of Vortex Dispersion
