# Supplementary material for: Spatially and spectrally resolved orbital angular momentum interactions in plasmonic vortex generators
Source: Light Sci Appl. 2019 Mar 20;8:33. doi: 10.1038/s41377-019-0136-z (PMC6425011; doi:10.1038/s41377-019-0136-z)
Supplement: Supplementary file 1 — Supplementary Materials [file 41377_2019_136_MOESM1_ESM.pdf]

Supplementary Information for

Spatially and spectrally resolved orbital angular momentum interactions in plasmonic vortex generators

Jordan A. Hachtel, Sang Yeon Cho, Roderick B. Davidson II, Matthew A. Feldman, Matthew F. Chisholm, Richard F. Haglund, Juan Carlos Idrobo, Sokrates T. Pantelides, Benjamin J. Lawrie

Effect of Channel Width on the Plasmon Response.

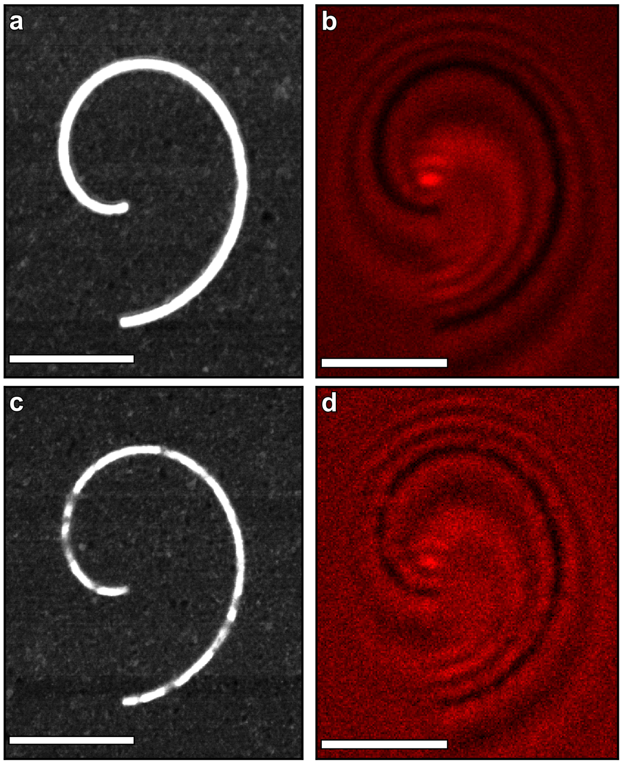

Figure S1. (a) Bright field (BF) BF scanning transmission electron microscope (STEM) image of a $d = 3\lambda_{SPP}$ with 100 nm channel from the main text. (b) 685 nm bandpass (BP) filtered photomultiplier tube (PMT) cathodoluminescence (CL) image of the channel shown in Figure S1a (same image as Figure 3c). (c) and (d) are equivalent images to (a) and (b) for a slit width of 75 nm. Scale bars 2 μm.

The effect of channel width on the plasmon vortex was investigated. A bright field (BF) scanning transmission electron microscope (STEM) image (Figure S1a) and a 685 nm band-pass (BP) filtered photomultiplier tube (PMT) cathodoluminescence (CL) image (Figure S1b) of a $d=3\cdot\lambda_{SPP}$ structure with a channel width of 100 nm (same as examined in the main text). We investigated if thinner channels could achieve higher coupling efficiency of the CL to SPPs. Figures S1c and S1d show the BF and 685 nm BP PMT-CL images of $d=3\cdot\lambda_{SPP}$ structure with a 75 nm wide channel. As can be seen from the BF, the channel is filled at many different locations, due to redeposition of silver during the focused ion-beam milling. Figure S1d shows that the plasmon vortex is less pronounced. The 100 nm channel width exhibited minimal redeposition, and so was used for all subsequent experiments.

Hyperspectral response of spiral channel

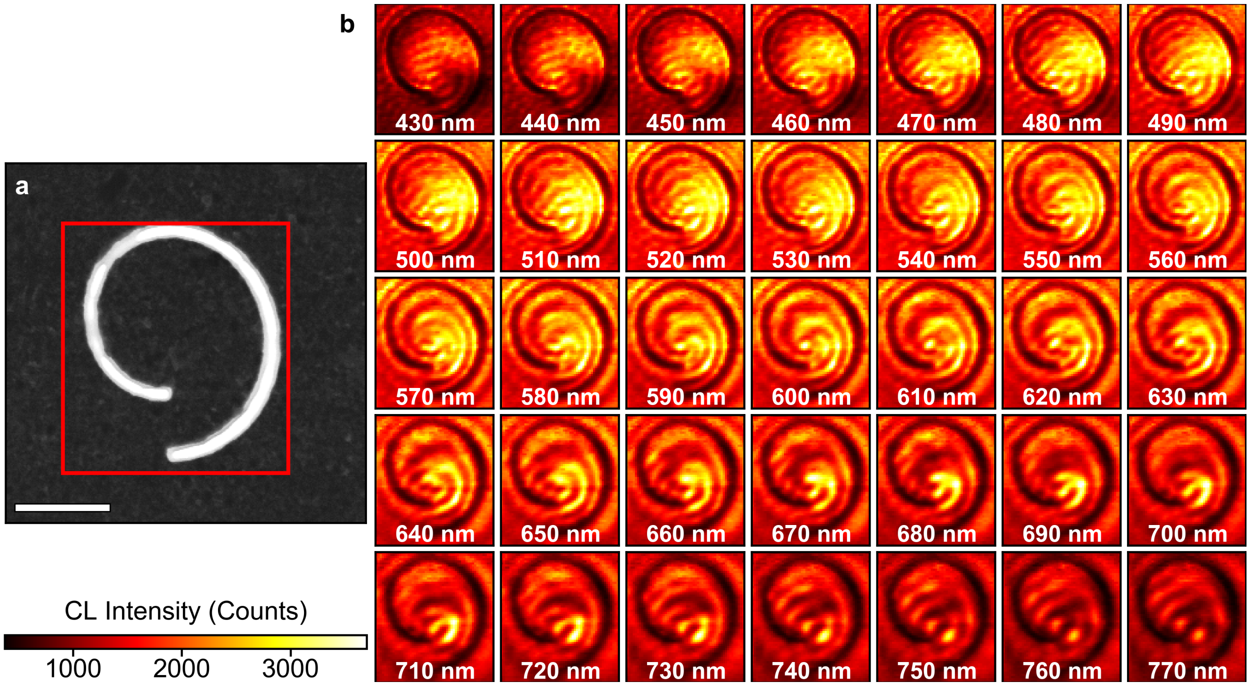

Figure S2. (a) BF-STEM image of the spiral channel shown in Figure S2a and S2b as well as Figure 2 of the main text. Scale bar 1 µm. (b) ALL frames of the SI shown in Fig. 1 in the main text in 10 nm wavelength bins between 430 nm and 770 nm.

In the main text, (Fig. 2) a spectrum image detailing the full spectral response of a plasmonic vortex is shown in 10 nm bins at intervals of 20 nm through the acquired range. Figure S2a shows the BF-STEM image of the spiral channel and the Figure S2b shows the SI from the main text except with all frames of the SI. It can be seen that both far below and far above the OAM wavelength of 660 nm, the CL response is dominated by linear interference fringes. However, for a bandwidth spanning 540-660 nm, the evidence of the plasmonic vortex wavefront emerges in the form of interlocking spiral arms (shown more clearly in Fig. S3). These spiral arms are the near-field structure of the plasmonic vortex discussed in the paper. The broad excitation of the vortex is due to (a) the fact that plasmon excitations are generally quite broadband excitations, and (b) the relatively high thickness of the Ag film (100 nm).

Characteristic CL Double Spiral Vortex Profile

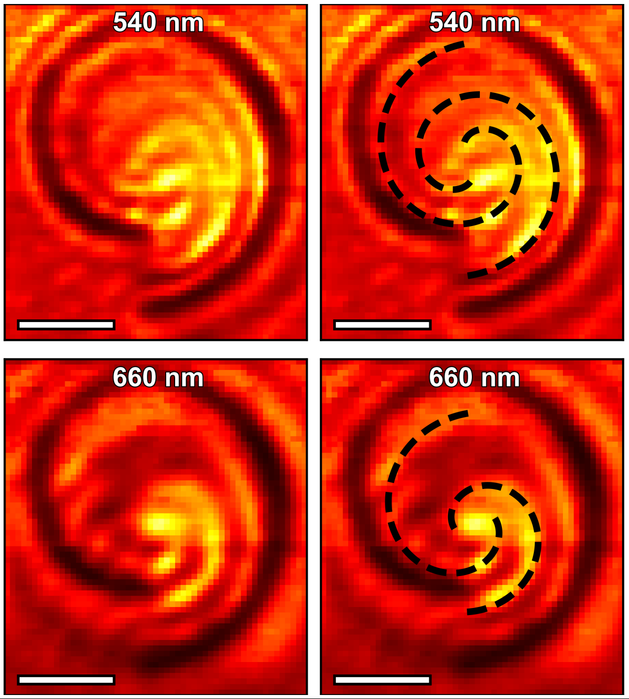

Figure S3. (Top Left) CL Profile of plasmonic vortex at 540 nm. (Top Right) 540 nm CL profile annotated with two Archimedean spirals matched to high intensity CL regions. (Bottom Left) and (Bottom Right) Non-annotated and double-spiral annotated images of 660 nm CL profile. Scale bars 1 μm.

In the main text, (Fig. 2), and in the supplementary information (Fig. S2), the CL-SI is shown with frame-by-frame wavelength slices. In both, we define the vortex to be resolved from wavelengths spanning from approximately 540 nm to 660 nm. Figure S3 demonstrates specifically how we define the bins where the vortex is resolved: the presence of two spiral arms of high intensity propagating out from the origin of the spiral channel.

The 20-nm wide CL-wavelength bins are shown for 540 nm (top) and 660 nm (bottom). On the right-hand panels, we have superimposed Archimedean spirals (dashed lines) on the image to demonstrate the orientation of the high CL-intensity arms, while the left-hand panels show only the CL intensity as a reference.

At both wavelengths it can clearly be seen that the high intensity arms match closely to the spiral annotations, demonstrating the spiral character of the plasmonic vortex. However, there are deviations from the spiral annotations. For instance, a 'fork' can be seen on the right-hand side of the 540 nm CL profile, indicating that the vortex is convolved with other types of plasmonic effects. Similarly, on the left-hand side of the 660 nm CL profile it can be seen that the spiral CL is convolved with some type of linear interference. By examining the wavelengths above 680 nm in Fig. 2 and Fig. S2 it can be seen that this linear interference eventually becomes the dominant signal. These irregularities are likely related to the 3D nature of the 100-nm-thick silver film, or possibly due surface roughness and channel roughness in the sample (which was shown to affect the CL vortex profile in Fig. S1). However, by comparing this region to the spiral arm it can be seen that there is a spiral path that matches the CL throughout the spiral, and the fork does not actually divert the spiral arm of the plasmonic vortex, so much as it expresses itself as additional contrast on top of the pure-vortex profile.

Effect of Arm Separation on Topological Charge of Plasmonic Vortex

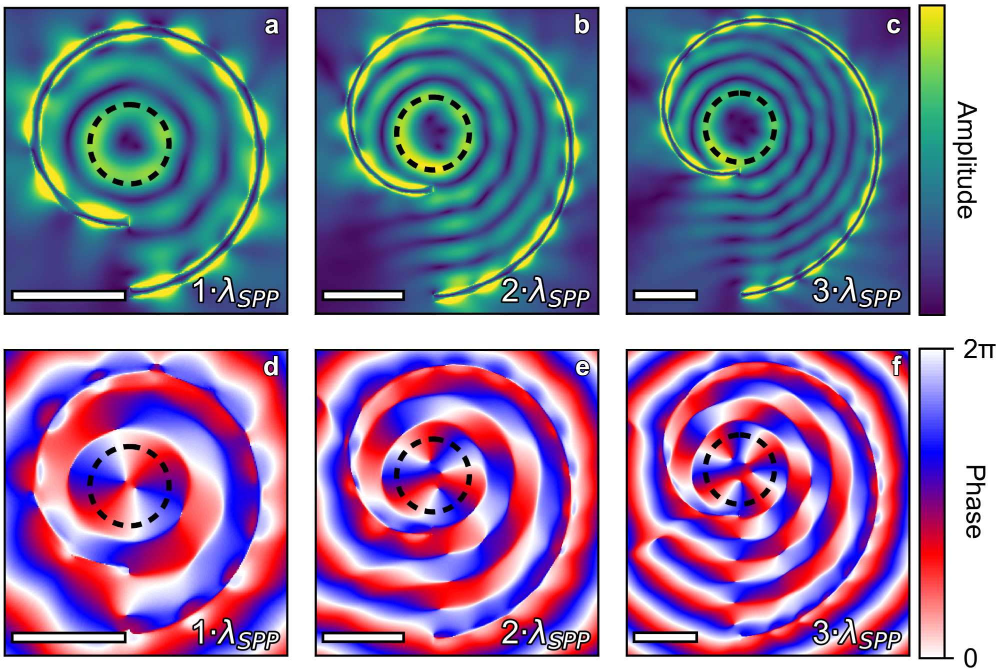

Figure S4. (a-c) FDTD simulations of the near-field plasmon amplitude for d=1 λ_{SPP}, 2 λ_{SPP}, and 3 λ_{SPP}, in response to a left-hand circularly polarized plane wave. (d-f) Corresponding phase plots.

For a plasmonic vortex in an Archimedean nanospiral, the topological charge is determined by the ratio of the arm separation to SPP wavelength. The effect is demonstrated here via FDTD simulations of the near-field response for d=1 λ_{SPP}, 2 λ_{SPP}, and 3 λ_{SPP} structures, like those found in Figure 4 of the main text, showing the response to a left-hand circularly polarized (LCP) plane wave at the free-space OAM wavelength of 660 nm. The LCP plane wave excitation also carries angular momentum, so the vortices observed in Fig. S4a and S4d are for a ℓ=2 vortex, for S4b and S4e ℓ=3, and for S4c and S4f ℓ=4.

In the plasmon amplitude plots shown in Figure S4a-c, the only effect is the increased size of the null-region of the plasmon vortex which does not provide any information on the topological charge. But for the phase plots in Figure S4d-f, the phase singularity at the origin changes significantly between the three structures. If one superimposes a dashed circle around the null region from the amplitude plots to better see the center of the plasmonic vortex and traces the total change in phase along the circumference, it can be seen that the ℓ=2 vortex has a phase shift of 4π, while the ℓ=3 has a phase shift of 6π, and the ℓ=4 has a phase shift of 8π. These integer increases in the phase singularity of the vortex demonstrate that the topological charge of the structure depends directly on the relationship between the arm separation and the SPP wavelength.
